# Increased CD4^+^CD8^+^ Double-Positive T Cell in Patients with Primary Sjögren's Syndrome Correlated with Disease Activity

**DOI:** 10.1155/2021/6658324

**Published:** 2021-05-14

**Authors:** Sen Wang, Han Shen, Bing Bai, Jia Wu, Junjun Wang

**Affiliations:** ^1^Department of Clinical Laboratory Medicine, Jinling Hospital, Medical School of Nanjing University, Nanjing 210002, China; ^2^Department of Clinical Laboratory Medicine, Nanjing Drum Tower Hospital, Medical School of Nanjing University, Nanjing 210008, China

## Abstract

Primary Sjogren's syndrome (pSS) is an autoimmune disease that invades lacrimal glands, salivary glands, and other exocrine glands, but its pathogenic mechanism is still unclear. CD4^+^CD8^+^ double-positive T (DPT) cells have been discovered in recent years to play an important role in autoimmune diseases and viral infections, but the frequency and significance of DPT in primary Sjogren's syndrome are still unclear. This study detected the frequency of DPT in the peripheral blood of patients with pSS and detected the clinical indicators and cytokines in patients. We then analyzed the correlation between DPT and clinical indicators, cytokines, and disease activity scores. The results showed that the peripheral DPT frequency of pSS patients was significantly higher than that of healthy controls. The peripheral DPT frequency was negatively correlated with ESR, IgA, and IgG, and peripheral DPT frequency was positively correlated with anti-inflammatory cytokine IL-10. Analysis of DPT and pSS disease activity scores found that DPT frequency had a negative correlation with ESSDAI and SSDAI. This study suggests that peripheral DPT may play a protective role in pSS. The frequency of peripheral DPT cells can be used as an indicator for disease activity. Regulating the expression of peripheral DPT cells is expected to become a new strategy for treatment of pSS.

## 1. Introduction

Primary Sjogren's syndrome (pSS) is a diffuse connective tissue disease characterized by invasion of lacrimal glands, salivary glands, and other exocrine glands, lymphocyte infiltration, and presence of specific autoantibodies (anti-SSA/SSB) [1, 2]. The first symptoms of pSS are diverse and clinical manifestations are complex. The typical manifestations are dry mouth and dry eyes. The systemic manifestations may include fever, fatigue, and joint pain. pSS can also involve the respiratory system, blood system, digestive system, and nervous system, resulting in multiple system damage [3].

There are many abnormalities of immune function in pSS patients. The abnormal distribution of lymphocyte subsets and the imbalance of cytokines are important mechanisms for pSS [4]. T cells are a heterogeneous cell population that can be divided into CD8^+^ cells and CD4^+^ cells according to different cell surface differentiation antigens. Peripheral CD4^+^CD8^+^ double-positive T (DPT) cells were firstly identified more than 30 years ago [5, 6]. At first, researchers thought that the DPT cells were a group of nonfunctional cells. Blue et al. have reported a small amount of DPT cells in human peripheral blood but this group of cells did not attract enough attention from researchers [5–7]. However, studies in recent years have found that the DPT cells have increased proportion in some viral infections and autoimmune diseases and may participate in the progression of the disease [8–10]. Howe et al. found that the number of DPT cells in the peripheral blood of HIV-1 infected patients increased significantly. Peripheral DPT cells can constitutively express cell-killing-related molecules C1.7 and Perforin. Moreover, the DPT cells can also produce IFN-*γ* and TNF that play a cytotoxic effect in HIV-1-infected patients [11]. Studies have also shown that there is a high proportion of DPT cells in the skin tissue of patients with systemic sclerosis. Compared with CD4 single-positive T cells, the DPT cells can produce more IL-4 and may be involved in the pathogenesis of systemic sclerosis [12]. The DPT cells inhibit the production of autoantibodies in patients with systemic lupus erythematosus (SLE) and may inhibit disease progression in SLE [13, 14].

Primary Sjogren's syndrome is a typical autoimmune disease but the proportion and role of DPT in Sjogren's syndrome are still unclear. This study detected the frequency of peripheral DPT and analyzed its correlation with clinical parameters, serum cytokines, and disease activity scores. This study also tried to explore the relationship between peripheral DPT in Sjogren's syndrome and disease activity and its significance in disease progression.

## 2. Materials and Methods

### 2.1. Patients and Controls

35 patients with pSS and 35 healthy controls were matched for sex and age and included in this study. pSS patients were admitted to the Nanjing Drum Tower Hospital from January 2019 to June 2019. The diagnosis of pSS was based on the 2002 American–European Consensus Group (AECG) classification criteria [15]. The gender, age, clinical manifestations, laboratory findings, treatment strategies, and other data of pSS patients were collected and analyzed. The disease activity was calculated using the EULAR Sjogren's syndrome disease activity index (ESSDAI) and Sjogren's syndrome disease activity index (SSDAI). The clinical characteristics of the pSS patients and healthy controls are summarized in Table 1. This study was approved by the medical ethics committee of the Nanjing Drum Tower Hospital of Nanjing University Medical School.

### 2.2. Flow Cytometry

50 *μ*L of peripheral blood from healthy controls and pSS patients were added into the flow cytometry tube. The cells were then stained with 5 *μ*L fluorescence-conjugated Abs: PerCP-Cy5.5-conjugated anti-CD3 Ab (BD Biosciences, USA), FITC-conjugated anti-CD4 (BD Biosciences, USA), and APC-conjugated anti-CD8 (BD Biosciences, USA). After mixing, the cells were incubated at room temperature for 15 minutes in the dark, and 1 mL of red blood cell lysis solution was added. After lysis at room temperature for 5 minutes, 3 mL of staining buffer (PBS containing 2% FBS) was added into the cells, followed by washing twice at 1500 rpm for 5 minutes. Flow cytometry was then used for detection. BD Cellquest software was used to obtain data by circling out CD3^+^ cells on the forward scattered light (FSC) and side scattered light (SSC) dot plots. Proportion of CD4/CD8 double-positive T cells to CD3^+^ cells was further analyzed. Flow data analysis and graphing used Flowjo software (Tree Star, USA).

### 2.3. ELISA

The concentrations of IL-6/TNF/IFN-*γ*/IL-10/IL-12 in the serum of healthy controls and pSS patients were determined by an ELISA kit (BD Biosciences, USA) following the manufacturer's protocols. Serum IFN-*α* was measured using the human IFN-alpha ELISA Kit (R&D Systems, USA) according to the manufacturer's protocols. An ELISA microplate reader was used to measure the absorbance at 450 nm.

### 2.4. Statistical Analysis

All analyses were performed using the GraphPad Prism version 5 (USA). The data are expressed as the media with interquartile range and compared by the Mann-Whitney *U* test. The correlation between DPT cells and clinical parameters of patients with pSS was measured by the Pearson correlative coefficient. *P* value < 0.05 was considered statistically significant.

## 3. Results

### 3.1. Increased Frequency of Peripheral DPT Cells in Patients with pSS

**Figure 1:**
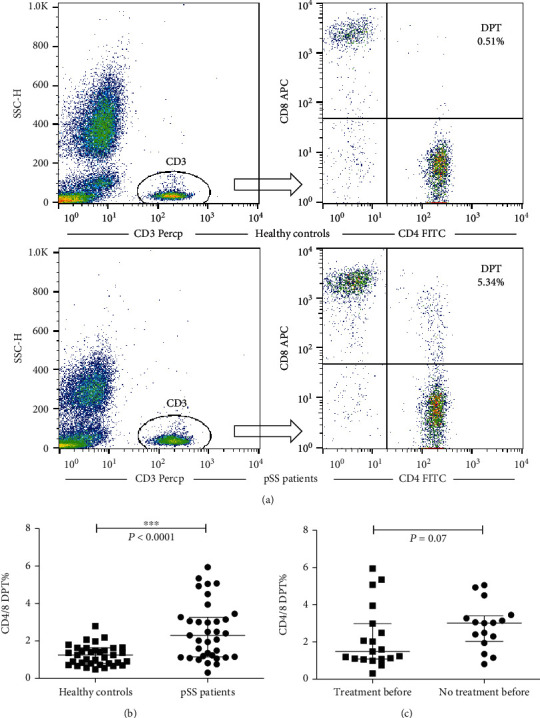
The frequency of peripheral DPT cells was increased in patients with pSS. (a) CD3^+^ cells were gated, and representative data of DPT were shown for healthy controls (upper panel) and pSS patients (lower panel). (b) Summary data of the frequency of peripheral DPT cells were shown for healthy controls in comparison with pSS patients. (c) Summary data for frequency of peripheral DPT cells were shown for patients receiving or not receiving immunosuppressive treatment before admission. Each dot represents one individual donor. The Mann-Whitney *U* test was used for comparison between the two groups. The error bars represent the median with interquartile range.

To study the frequency and clinical significance of DPT cells in pSS patients, we used flow cytometry to detect the frequency of DPT cells in the peripheral blood CD3-positive cells of pSS patients and healthy controls.  Figure 1(a) shows the gating strategy and frequency of DPT cells in typical pSS patient and healthy controls. Results showed that the proportion of DPT cells in CD3^+^ cells in pSS patients was significantly higher than that in healthy controls (Figure 1(b)). Among the 35 patients with pSS, 16 had not received immunosuppressive therapy before admission. Our results showed that there was no significant difference in DPT frequency between patients receiving immunosuppressive therapy and those who did not receive immunosuppressant therapy before admission (Figure 1(c)). We analyzed the relationship between peripheral DPT frequency and autoantibodies. The results showed that there was no significant difference in DPT frequency between anti-SSA/SSB positive patients and negative patients. In addition, there was no correlation between the frequency of peripheral DPT and the titer of antinuclear antibodies (Figure S1).

### 3.2. Correlation between Peripheral DPT Cells and Clinical Parameters

**Figure 2:**
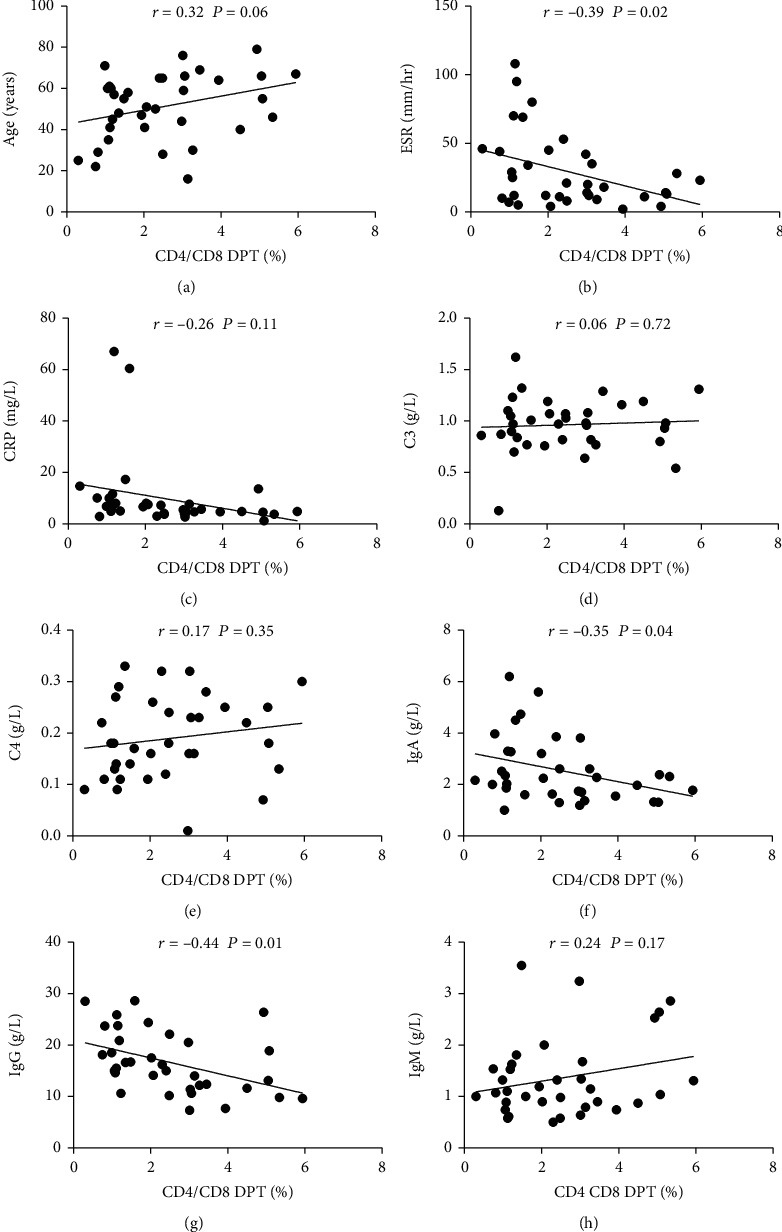
Correlation between frequency of peripheral DPT cells and clinical parameters. (a–h) Correlation of peripheral DPT cells with age, ESR, CRP, C3, C4, IgA, IgG, and IgM.

In order to clarify the clinical significance of increased peripheral DPT cell frequency in pSS patients, we detected the relevant chemical and immunological parameters in the patient's serum and analyzed the correlation between peripheral DPT cell frequency and these clinical parameters. The results showed that the frequency of peripheral DPT cells did not increase with age of the patient (Figure 2(a)). Correlation analysis showed that peripheral DPT frequency was negatively correlated with ESR, but not statistically correlated with CRP, C3, and C4 (Figures 2(b)–2(e)). The level of immunoglobulin in patients with pSS was higher than that in normal people and was related to the activity of the disease. Therefore, we also analyzed the correlation between frequency of peripheral DPT and immunoglobulin. We found that the frequency of peripheral DPT cells had no correlation with IgM, but there was negative correlation with the levels of IgA and IgG (Figures 2(f)–2(h)).

### 3.3. The Level of Cytokines Was Elevated in the Serum of pSS Patients

**Figure 3:**
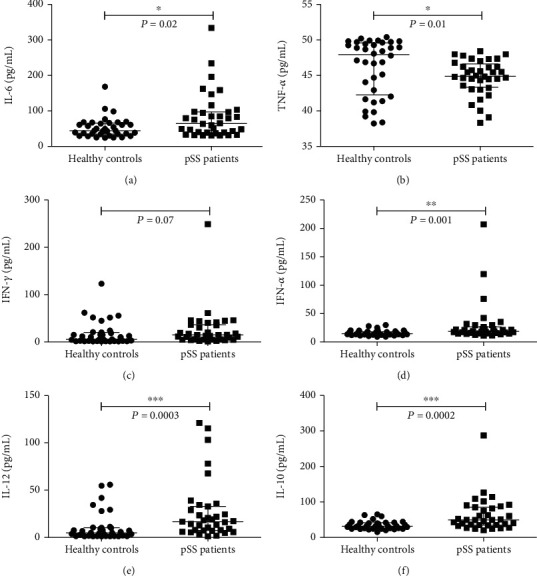
Comparison of cytokine levels between healthy control group and pSS patient group. (a–f) The levels of IL-6, TNF-*α*, IFN-*γ*, IFN-*α*, IL-12, and IL-10 were detected in healthy controls and pSS patients. Each dot represents one individual donor. The Mann-Whitney *U* test was used for comparison between the two groups. The error bars represent the median with interquartile range.

Cytokines are one of important ways for T cells to function. We detected the expression levels of various cytokines in the serum of pSS patients and healthy controls using ELISA. It was found that the levels of IL-6, TNF-*α*, IFN-*α*, IL-12, and IL-10 in pSS patients were significantly higher than in healthy controls (Figures 3(a)–3(f)). This was consistent with increased serum cytokines in pSS patients in previous reports [16].

### 3.4. Frequency of Peripheral DPT Cells Was Positively Correlated with Level of IL-10

**Figure 4:**
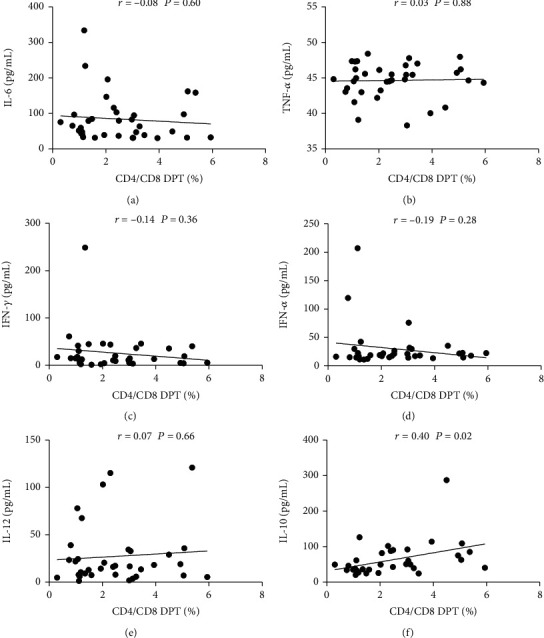
Correlation analysis between the frequency of peripheral DPT cells and IL-6, TNF-*α*, IFN-*γ*, IFN-*α*, IL-12, and IL-10 (a–f).

In order to further analyze the possible role of peripheral DPT cells in pSS patients, we analyzed the correlation between frequency of peripheral DPT cells and serum cytokines. The results showed that the peripheral DPT frequency had no correlation with IL-6, TNF-*α*, IFN-*γ*, IFN-*α*, and IL-12 (Figures 4(a)–4(e)), but it had positive correlation with anti-inflammatory cytokine IL-10 (Figure 4(f)).

### 3.5. Frequency of Circulating DPT Cells Was Negatively Correlated with ESSDAI/SSDAI

**Figure 5:**
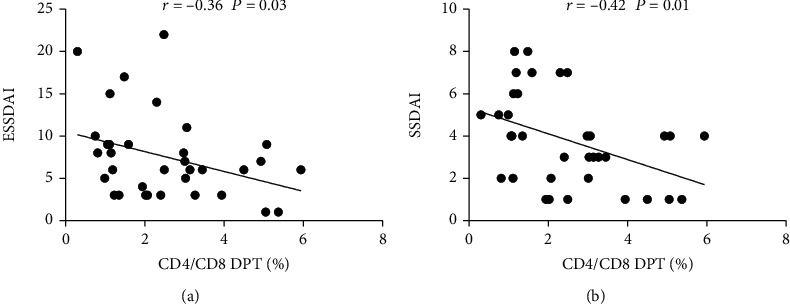
Correlation analysis between the frequency of peripheral DPT cells and ESSDAI, SSDAI (a, b).

ESSDAI and SSDAI are commonly used clinical pSS disease activity scores. In this study, patients were scored according to clinical symptoms and detection indicators when pSS patients were admitted to the hospital, and the correlation between ESSDAI/SSDAI scores and peripheral blood DPT levels was analyzed. As shown in Figure 5, the results from the study found that the peripheral blood DPT level of patients with pSS had a negative correlation with ESSDAI and SSDAI scores, suggesting that the DPT cells may play a protective role in the pathogenesis and progression of pSS.

## 4. Discussion

Sjogren's syndrome is a chronic inflammatory autoimmune disease that can affect multiple systems. It mainly invades exocrine glands and can also involve nonexocrine gland tissues. Peripheral DPT cells are a group of cells that have been neglected, but have been found to function in viral infections and some autoimmune diseases in recent years. The frequency and role of peripheral DPT cells in patients with Sjogren's syndrome are currently unknown. This study detected the frequency of DPT cells in the peripheral blood of pSS patients and analyzed its correlation with clinical parameters. It was found that the frequency of DPT cells in pSS patients was significantly higher than that of healthy controls. Moreover, the frequency of DPT cells was negatively correlated with ESR, IgA, IgG, ESSDAI, and SSDAI, and positively correlated with IL-10 cytokine. This study also showed that the DPT cells were elevated in pSS patients and may have a protective effect on pSS patients.

During the development of T cells in the thymus, there is a stage of double positive for CD4 and CD8. However, due to negative and positive selection mediated by TCR, mature T cells entering the peripheral circulation are CD4 or CD8 single-positive cells [17]. Regarding the source of peripheral DPT cells, studies have suggested that single-positive T cells can be induced to become double-positive cells when stimulated in vitro. Blue and his colleagues isolated and purified CD4^+^CD8^−^ and CD4^−^CD8^+^ T cells in vitro. After stimulating these purified cells with ConA, they found that single-positive T cells can produce CD4/CD8 double-positive T cells [5]. Moreover, Paliard and his colleagues found that the addition of IL-4 in vitro can promote the de novo synthesis of CD8 receptors by purified CD4 single-positive T cells [18]. This process is completely dependent on the presence of IL-4. Both IL-10 and IL-4 are anti-inflammatory cytokines [19]. In this study, it was found that the frequency of peripheral DPT cells was positively correlated with IL-10. Therefore, IL-10 may also be one of the factors that induce single-positive T cells to differentiate into DPT cells. Since this study mainly focused on the clinical significance of increased DPT frequency in pSS patients, it did not further explore the source of DPT, which was a limitation of this study. Studies have shown that peripheral DPT cells highly express ab T cell receptors, CD45RO, and CCR7, and therefore belong to central memory T cells. The expression of HLA-DR and CD56 on peripheral DPT cells is higher than that of CD4 single-positive T cells, but DPT cells do not express iNKT markers [20]. Because the phenotype of DPT cells has been reported in the literature, the phenotype was not studied in this study.

Cytokines play an important role in the occurrence and development of pSS. This study tested a variety of cytokines including IL-6, TNF-a, IFN-*γ*, IFN-*α*, IL-12, and IL-10. Among them, IL-6, IFN-*α*, IL-12, and IL-10 were significantly higher than in the healthy controls, which is consistent with previous studies [16]. We analyzed the correlation between peripheral DPT cells and cytokines and found that the peripheral DPT cells were only positively correlated with IL-10 levels. IL-10 is an immunosuppressive cytokine that can mediate immune tolerance and tumor immune escape and also plays a key role in anti-inflammation. IL-10 can be produced by macrophages, Breg, and some T cells such as Treg [21, 22]. In this study, the level of IL-10 in the serum of patients with pSS was increased, and it was positively correlated with the level of peripheral DPT, suggesting that DPT may produce IL-10 which play the immunosuppression function. Whether DPT cells can directly produce IL-10 and play a role in inhibiting inflammation in pSS patients requires further research.

The functions of peripheral DPT cells may be different in different diseases. During HIV infection, the DPT cells were highly proliferative and active effector cells, and their proliferation and effect capabilities were stronger than single-positive T cells [23]. The frequency of CD4/CD8 DP T cells was significantly increased in melanoma. These DPT cells have a unique cytokine profile and have antitumor activity in vitro [24]. However, in a study of metastatic colorectal cancer, the DPT cells were thought to promote tumor growth or metastasis and downregulate the immune response to colorectal cancer [25]. DPT cells are thought to have immunosuppressive effects in autoimmune diseases. In systemic sclerosis, the DPT cells in the patient's skin tissue can produce high levels of IL-4 to exert immunosuppressive functions [12]. DPT cells are also present in the intestinal epithelial layer in inflammatory bowel disease, and can inhibit Th1-induced intestinal inflammation in an IL-10-dependent manner [26]. In SLE patients, the DPT cells are thought to inhibit the production of autoantibodies. However, in this study, we did not find a significant correlation between DPT cells and SSA/SSB/ANA in pSS patients, and more samples may need to be included to clarify this. In this study, the frequency of peripheral DPT in patients with pSS was increased, which was negatively correlated with ESR and IgG, which was positively correlated with anti-inflammatory cytokine IL-10 and was negatively correlated with disease activity score, thus suggesting that the peripheral DPT has immunosuppression function that plays a protective role in the occurrence and development of diseases.

In summary, the frequency of peripheral DPT in patients with pSS was significantly increased in this study. Correlation analysis with clinical parameters, cytokines, and disease activity scores indicated that the DPT cells have an immunosuppressive effect in patients with pSS. The DPT cells may thus be used as potential strategy in treating pSS or relieving inflammation symptoms.

## Figures and Tables

**Table 1:** Characteristics of study subjects.

	pSS patients	Health controls	*P*
Number (*n*)	35	35	—
Age (years)	55 (41-65)	57 (36-63)	0.71
Gender (male/female)	2/33	3/32	—
Disease duration (years)	2.50 (1.00-8.25)	—	—
Anti-SSA (+/−)	27/8	—	—
Anti-SSB (+/−)	19/16	—	—
Antinuclear antibodies (+/−)	25/10	—	—
ESSDAI score	6.00 (3.00-9.00)	—	—
SSDAI score	4.00 (2.00-5.00)	—	—
CRP (mg/L)	5.70 (4.60-8.00)	1.80 (1.50-2.20)	0.003
ESR (mm/h)	20 (11-44)	10 (6-14)	<0.001
C3 (g/L)	0.97 (0.82-1.10)	1.10 (0.94-1.21)	0.02
C4 (g/L)	0.18 (0.13-0.25)	0.23 (0.18-0.29)	0.007
IgG (g/L)	15.50 (11.60-20.90)	12.10 (11.40-12.60)	<0.001
IgA (g/L)	2.24 (1.63-3.28)	2.19 (1.49-2.55)	0.36
IgM, g/L	1.10 (0.87-1.63)	1.33 (1.04-1.95)	0.47
Immunosuppressive therapy (Y/N)	19/16	—	—

The data are expressed as the median (25% percentile-75% percentile). The Mann-Whitney *U* test was used for comparison between pSS patients and health controls.

## Data Availability

The data used to support the findings of this study are available from the corresponding authors upon request.

## Supplementary Materials

Supplementary Materials Figure S1: correlation between peripheral DPT frequency and autoantibodies. Comparison of peripheral DPT frequency between SSA/SSB positive and negative patients. The error bars represent the median with interquartile range (a). Correlation between peripheral DPT frequency and antinuclear antibody titer (b).
