# Supplementary material for: Increased CD4+CD8+ Double-Positive T Cell in Patients with Primary Sjögren's Syndrome Correlated with Disease Activity
Source: J Immunol Res. 2021 May 14;2021:6658324. doi: 10.1155/2021/6658324 (PMC8140824; doi:10.1155/2021/6658324)
Supplement: Supplementary Materials — Figure S1: correlation between peripheral DPT frequency and autoantibodies. Comparison of peripheral DPT frequency between SSA/SSB positive and negative patients. The error bars represent the median with interquartile range (a). Correlation between peripheral DPT frequency and antinuclear antibody titer (b). [file 6658324.f1.zip › SUPPLEMENTARY DESCRIPTION.docx]

SUPPLEMENTARY DESCRIPTION:

Figure S1: Correlation between peripheral DPT frequency and autoantibodies. Comparison of peripheral DPT frequency between SSA/SSB positive and negative patients. The error bars represent the median with interquartile range. (a). Correlation between peripheral DPT frequency and antinuclear antibody titer (b).
